# Epigenetics override pro-inflammatory PTGS transcriptomic signature towards selective hyperactivation of PGE_2_ in colorectal cancer

**DOI:** 10.1186/s13148-015-0110-4

**Published:** 2015-07-24

**Authors:** Inês Cebola, Joaquin Custodio, Mar Muñoz, Anna Díez-Villanueva, Laia Paré, Patricia Prieto, Susanna Aussó, Llorenç Coll-Mulet, Lisardo Boscá, Victor Moreno, Miguel A. Peinado

**Affiliations:** Institute of Predictive and Personalized Medicine of Cancer (IMPPC, Ctra Can Ruti, Cami de les Escoles, Badalona, 08916 Spain; Unit of Biomarkers and Susceptibility, Cancer Prevention and Control Program, Catalan Institute of Oncology (ICO), IDIBELL and CIBERESP, Hospitalet de Llobregat, Barcelona Spain; Instituto de Investigaciones Biomédicas Alberto Sols (CSIC-UAM), Madrid, Spain; Department of Clinical Sciences, Faculty of Medicine, University of Barcelona, Barcelona, Spain; Current address: Department of Medicine, Imperial College London, London, UK; Current address: Science for Life Laboratory, Department of Medical Biochemistry and Biophysics, Karolinska Institutet, Stockholm, Sweden

**Keywords:** DNA methylation, Gene expression, COX pathway, Prostanoids, Inflammation, Prostaglandins

## Abstract

**Background:**

Misregulation of the PTGS (prostaglandin endoperoxide synthase, also known as cyclooxygenase or COX) pathway may lead to the accumulation of pro-inflammatory signals, which constitutes a hallmark of cancer. To get insight into the role of this signaling pathway in colorectal cancer (CRC), we have characterized the transcriptional and epigenetic landscapes of the PTGS pathway genes in normal and cancer cells.

**Results:**

Data from four independent series of CRC patients (502 tumors including adenomas and carcinomas and 222 adjacent normal tissues) and two series of colon mucosae from 69 healthy donors have been included in the study. Gene expression was analyzed by real-time PCR and Affymetrix U219 arrays. DNA methylation was analyzed by bisulfite sequencing, dissociation curves, and HumanMethylation450K arrays. Most CRC patients show selective transcriptional deregulation of the enzymes involved in the synthesis of prostanoids and their receptors in both tumor and its adjacent mucosa. DNA methylation alterations exclusively affect the tumor tissue (both adenomas and carcinomas), redirecting the transcriptional deregulation to activation of prostaglandin E_2_ (PGE_2_) function and blockade of other biologically active prostaglandins. In particular, *PTGIS*, *PTGER3*, *PTGFR*, and *AKR1B1* were hypermethylated in more than 40 % of all analyzed tumors.

**Conclusions:**

The transcriptional and epigenetic profiling of the PTGS pathway provides important clues on the biology of the tumor and its microenvironment. This analysis renders candidate markers with potential clinical applicability in risk assessment and early diagnosis and for the design of new therapeutic strategies.

**Electronic supplementary material:**

The online version of this article (doi:10.1186/s13148-015-0110-4) contains supplementary material, which is available to authorized users.

## Background

There is strong evidence associating inflammation with cancer [1–3]. Risk factors such as tobacco smoke, high-fat diet, and chronic infection are correlated with chronic inflammation [4], and the tumor-microenvironment itself has an intrinsic inflammatory component [1, 2]. Prostanoids are signaling molecules with important pro- and anti-inflammatory roles synthesized from arachidonic acid via the PTGS (prostaglandin endoperoxide synthase, also known as cyclooxygenase or COX) pathway. Deregulation of the enzymes of this pathway during inflammatory processes, including tumorigenesis, results in abnormal levels of the different prostanoids [5–7].

The association of genetic polymorphisms in PTGS pathway genes and colorectal cancer risk and survival [8–11] supports its involvement in the etiology of the disease. PTGS2 (also known as COX-2), one of the key enzymes of this pathway, is frequently overexpressed in colorectal tumors, which results in overproduction of the downstream metabolite prostaglandin E_2_ (PGE_2_). Both, PTGS2 expression and PGE_2_ levels have been shown to correlate with metastasis and poor prognosis in colorectal cancer patients [12, 13, 7, 14–16]. A recent study has also highlighted the participation of PGE_2_/PTGS2 signaling during development of chemoresistance [17].

Aspirin and other non-steroidal anti-inflammatory drugs (NSAIDs) are able to inhibit cyclooxygenase activity and have been shown to reduce the risk and improve the outcome of colorectal cancer (CRC) and other gastrointestinal tumors [18–23]. However, their prescription for chemoprevention of colorectal cancer is restricted to high-risk individuals due to an associated increased risk of hemorrhagic strokes and gastrointestinal complications [24, 22]. PTGS2-specific inhibitors (coxibs) show lower gastrointestinal toxicity, but an increased risk of cardiovascular complications [25, 22].

Transformation of the colorectal tissue is characterized by the successive acquisition of genetic and epigenetic alterations that confer advantageous traits for tumorigenesis initiation and cancer progression [26]. Among these, DNA methylation alterations are known to be involved [27–30]. Whereas DNA hypomethylation of repetitive regions and oncogenes increases genomic instability and facilitates aberrant re-expression of imprinted genes, promoter CpG island DNA hypermethylation results in the acquisition of a repressed chromatin state and consequent gene silencing. This process is known to underlie the silencing of several tumor-suppressor genes in cancer, including APC and p16, which contribute to the acquisition and maintenance of an oncogenic state [29, 30].

Numerous components of this pathway have been found deregulated by DNA hypermethylation in cancer (reviewed in [31]). However, to our knowledge, no study has addressed how DNA methylation of multiple genes may affect the overall prostanoid production in the transformed colorectal tissue, nor in other types of cancer. Here, we have investigated transcriptional and epigenetic profiles of the PTGS pathway in four series of colorectal cancer patients (Additional file 1: Table S1). We report a global deregulation of this pathway in both the colonic mucosae and the tumor and pinpoint a set of features that might be of value as new diagnostic markers and/or as therapeutic targets in colorectal cancer patients.

## Results

### Expression profiling of the PTGS pathway in colorectal tissue

Previous reports have shown that the PTGS pathway is frequently deregulated in a number of cancers reviewed in [31]. While some of its major pathology-related features have already been studied in depth, including the overexpression of *PTGS2* during inflammation and tumorigenesis [32, 6], the complete picture of the regulatory state of this pathway in CRC remains elusive.

**Fig. 1:**
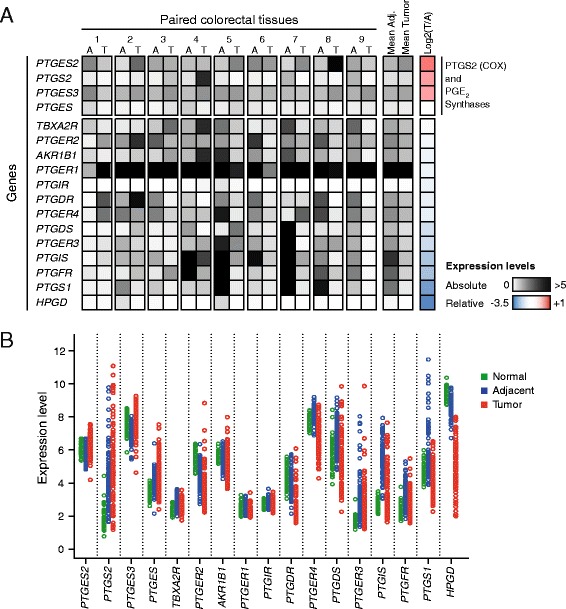
PTGS pathway expression profiling in colorectal cancer. **a** Preliminary analysis of a set of nine colorectal tumors by quantitative real-time PCR reveals overall imbalance of gene expression regulation in comparison with adjacent normal colonic mucosae, being most of the genes downregulated. Exceptionably, the expression of genes responsible for PGE_2_ biosynthesis is maintained or increased. Absolute expression levels were calculated by the delta Ct method (see “Methods” section). Relative expression corresponds to tumor/adjacent mucosa ratio. **b** Microarray analysis performed on a cohort of 98 CRC patients (both tumor and adjacent mucosae) and 50 matched healthy donors. As in **a**, most genes are deregulated, with marked differences not only between tumors and their adjacent normal tissues, but also between normal tissues from patients and healthy individuals

To gain a first glimpse at the changes the PTGS pathway undergoes during colorectal tumorigenesis, we analyzed gene expression levels in a series of nine CRC tumors and adjacent mucosae. Even though the samples presented heterogeneous expression profiles, our results show that the transcriptional profile of the PTGS pathway is markedly altered during tumorigenesis, presenting downregulation of many genes in the majority of tumors (Fig. 1a). On the other hand, we observed overall increased expression of PGE_2_ synthases (especially of *PTGES2* and *PTGES3*) (gene nomenclature is shown in Additional file 1: Table S2).

We then extended our study by applying microarray expression analysis to a cohort of 98 CRC patients (Table 1). In order to identify possible disease-related features in the adjacent tissues of patients, this analysis included the colonic tissues from a control cohort comprising 50 healthy individuals. Following the trend registered in our test cohort, we observed frequent downregulation of eight genes and recurrent overexpression of all PGE_2_ synthases (*PTGES*, *PTGES2* and *PTGES3*, Tukey’s HSD test *P* < 0.0001) in CRC tumors (Fig. 1b, Table 2). In accordance with our initial analysis and previous reports [33, 34], *PTGS2* showed a heterogeneous pattern, being only overexpressed in a subset of tumors (Fig. 1b, Table 2, Tukey’s HSD test *P* = 0.0091). PTGS2 protein analysis revealed an equally variable expression pattern (data not shown).

**Table 1:** Baseline characteristics of healthy donors and CRC patients from the *Colonomics* study

Healthy donors (*n* = 50)	
Gender	
Male	27 (54 %)
Female	23 (46 %)
Median age (years, range)	63 (25–88)
Site	
Right	27 (54 %)
Left	23 (46 %)
Cases (*n* = 98)	
Gender	
Male	71 (72.4 %)
Female	27 (27.6 %)
Median age (years, range)	71 (43–87)
Site	
Right	38 (38.8 %)
Left	60 (61.2 %)
Stage	
II A	90 (91.8 %)
II B	8 (8.2 %)
Recurrence	
No relapse	76 (77.6 %)
Relapse	22 (22.4 %)
Follow-up, median time (months, range)	67.8 (24.8–136.9)

**Table 2:** Summary table with *P* values for the comparisons of tumors and normal mucosae from patients and healthy donors from the *Colonomics* study

	Gene expression			DNA methylation		
	N vs. A	A vs. T	ANOVA	N vs. A	A vs. T	ANOVA
*AKR1B1*	0.9482	0.0171	0.007	0.7758	<0.0001	<0.0001
*HPGD*	<0.0001	<0.0001	<0.0001	–	–	0.076
*PTGDR*	0.2583	<0.0001	<0.0001	–	–	0.1083
*PTGDS*	<0.0001	<0.0001	<0.0001	–	–	0.0837
*PTGER1*	0.7856	0.0341	0.0414	0.8720	0.0052	<0.0001
*PTGER2*	<0.0001	<0.0001	<0.0001	–	–	0.3878
*PTGER3*	<0.0001	0.0037	<0.0001	0.8859	<0.0001	<0.0001
*PTGER4*	0.0005	<0.0001	<0.0001	–	–	0.9691
*PTGES*	<0.0001	<0.0001	<0.0001	0.6580	0.0222	0.0147
*PTGES2*	0.0158	<0.0001	<0.0001	–	–	0.6959
*PTGES3*	<0.0001	<0.0001	<0.0001	–	–	0.0545
*PTGFR*	<0.0001	<0.0001	<0.0001	0.7811	<0.0001	<0.0001
*PTGIR*	0.0005	0.0086	0.0003	0.9245	<0.0001	<0.0001
*PTGIS*	<0.0001	<0.0001	<0.0001	0.9392	<0.0001	<0.0001
*PTGS1*	<0.0001	<0.0001	<0.0001	0.0118	<0.0001	<0.0001
*PTGS2*	<0.0001	0.0091	<0.0001	0.9866	<0.0001	<0.0001
*TBXA2R*	<0.0001	<0.0001	<0.0001	0.0362	0.0029	<0.0001

ANOVA test was applied to determine significant differences among the three types of sample. Genes with significant differences (*P* < 0.05) were further evaluated with Tukey’s range test for differences of normal colonic mucosae from healthy donors (N) versus adjacent tissue from CRC patients (A), and of adjacent mucosae (A) versus CRC tumors (T)

Noteworthy, we observed significant alterations in the transcriptional profile of mucosae adjacent to tumors in comparison to normal mucosae obtained from healthy donors, with many genes showing a rebound effect during the tumorigenic process. Specifically, *PTGES2* and *PTGES3* tended to be downregulated in adjacent mucosae (Tukey’s HSD test *P* = 0.0158 and *P* < 0.0001, respectively), but overexpressed in tumors (*P* < 0.0001 for both) (Fig. 1b, Table 2). The opposite trend was observed with *PTGER4*, *PTGDS*, *PTGER3*, *PTGIS*, *PTGFR*, and *PTGS1* genes, which showed overexpression in the adjacent non-tumor tissue, followed by downregulation in the tumor. *PTGS2* was found significantly overexpressed in adjacent mucosae from patients (Tukey’s HSD test *P* < 0.0001). The observed trends were even more evident when we examined a panel of five CRC cell lines (Additional file 1: Figure S1A).

Overall, these results demonstrate an abnormal behavior of most of the genes of the PTGS pathway not only in tumors, but also in the non-tumor adjacent tissue of CRC patients. Even though the altered expression of many genes is maintained or even exacerbated in tumors, a subset overturns its deregulation, reversing the expression levels in the cancer tissue to either upregulation (i.e., *PTGES3*) or a strong downregulation (i.e., *PTGIS*).

### DNA methylation profiling of the PTGS pathway in colorectal tissue

Previous reports from our lab and others have shown that PTGS pathway genes can undergo epigenetic silencing during cancer, in particular through promoter-associated-CpG island DNA methylation [31, 35]. For this reason, we decided to investigate whether promoter methylation could be the mechanism responsible for the repression of these genes in CRC, having combined three independent sets of samples and two DNA methylation detection methods.

**Fig. 2:**
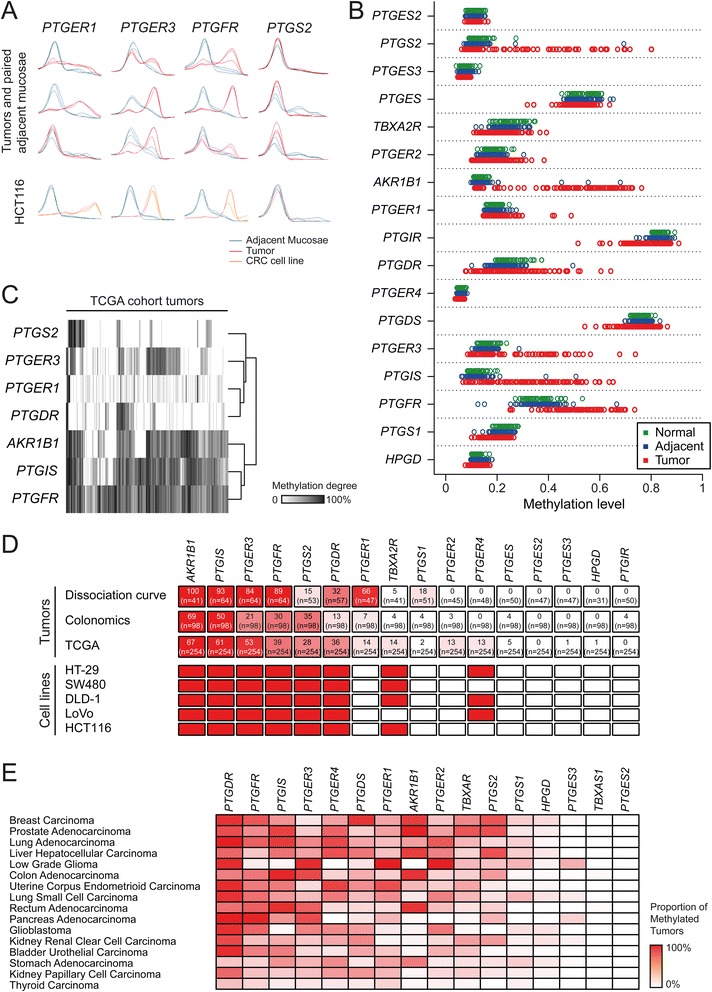
PTGS pathway DNA methylation profiling in colorectal cancer. **a** Dissociation curve analysis of CRC patients and cell lines. A total of 64 patients (tumor and adjacent mucosae pairs) and five cell lines were analyzed with this method. Cell lines were compared to an unmethylated normal tissue. **b** DNA methylation beta values quantified with InfiniumMethylation450K arrays for a cohort of 98 CRC patients (both tumor and adjacent mucosae) and 50 matched healthy donors. Unlike gene expression, adjacent normal tissue of CRC patients displays normal methylation profiles compared with the mucosae from healthy donors, while tumors show a large number of abnormalities. **c** Unsupervised clustering analysis of the DNA methylation levels of 254 CRC tumors from in the Cancer Genome Atlas (TCGA). AKR1B1, PTGIS, and PTGER3 were the genes exhibiting the highest rates of hypermethylation in CRC. **d** DNA methylation analysis by dissociation curve and InfiniumMethylation450K array (*Colonomics* study and TCGA) provide similar results. *White* to *red color scale* represents the minimum and maximum percentage of hypermethylated tumors, respectively. Methylation status of the PTGS pathway genes in a panel of six colorectal cancer cell lines is represented. Genes frequently methylated in CRC tumors are also methylated in the analyzed cell lines. *Red* represents methylation in >75 % of the CpG sites. **e** Proportion of tumors with DNA methylation of the PTGS pathway genes in different cancer types. Data were obtained from TCGA

Dissociation curve analysis was applied to detect changes in the DNA methylation content of PTGS pathway genes in 64 CRC patients, together with five CRC cell lines (Fig. 2a). In addition, we assessed the DNA methylation profiles of 98 CRC patients (tumor and adjacent mucosa pairs) and 50 healthy individuals with the InfiniumMethylation450K platform (Fig. 2b, Table 2). Both methodologies revealed the same trends, being the *AKR1B1*-, *PTGIS*-, *PTGFR*-, and *PTGER3*-associated CpG islands, the ones altered at a higher rate (Fig. 2d). Similar results were observed in the publically available datasets from the TCGA (Fig. 2c, d).

Next, we wondered whether the methylation abnormalities occurred early in tumor progression. We tackled this issue by analyzing data available from a recent study in which DNA methylation profiles were also analyzed with the same platform in normal colonic mucosae, adenomas, and carcinomas [36]. Interestingly, the methylation profiles of adenomas mimicked those of carcinomas, indicating the contribution of DNA methylation alterations early in tumorigenesis (Additional file 1: Figure S2). Once again, normal mucosa from CRC patients showed no alterations when compared with healthy individuals, confirming our previous observations with the Colonomics series.

We also analyzed the DNMT double-knockout cell line HCT116-DKO [37], which presented reconstitution of the expression of most of these genes, supporting our hypothesis of an epigenetic silencing mechanism—DNA methylation—behind the observed transcriptional downregulation in CRC (Additional file 1: Figure S1B).

### Profiling of the PTGS pathway in colorectal cancer progression

DNA methylation is not the only possible mechanism responsible for gene downregulation and silencing. For this reason, we further interrogated the TCGA database for DNA sequence alterations in PTGS pathway genes. This analysis all included all indels, large deletions and amplifications detected in PTGS pathway genes in CRC tumors. Overall, approximately a quarter of the analyzed tumors presented a mutation in at least one of the genes, but when analyzed individually, none of the genes revealed a high frequency of deleterious mutations in colorectal tumors. Furthermore, no deleterious mutations have been detected in any of the PGE_2_ synthases, neither in the receptors *PTGER2* and *TBXA2R* (Additional file 1: Figure S3A). Interestingly, although the observed mutation rates are very low, there still seems to be significant poor prognosis associated with mutations in *PTGFR*, one of the genes most predominantly hypermethylated and downregulated in colorectal tumors (Additional file 1: Figure S3B).

Considering that primary tumors from closely related tissues tend to share common molecular signatures, we postulated that the methylation patterns found in colorectal cancer could be also found in other cancer types. Indeed, not only colorectal, but also liver, stomach, and pancreas tumors present high rates of hypermethylation of *PTGIS*, *AKR1B1*, *PTGER3*, and *PTGFR* (Fig. 2e). Our data together with data from TCGA suggest that promoter-associated CpG island DNA methylation is the major mechanism involved in the deregulation of the PTGS pathway in colorectal and other types of cancer in the gastrointestinal tract.

### Gene expression profiles of normal mucosae distinguish patients from healthy donors

**Fig. 3:**
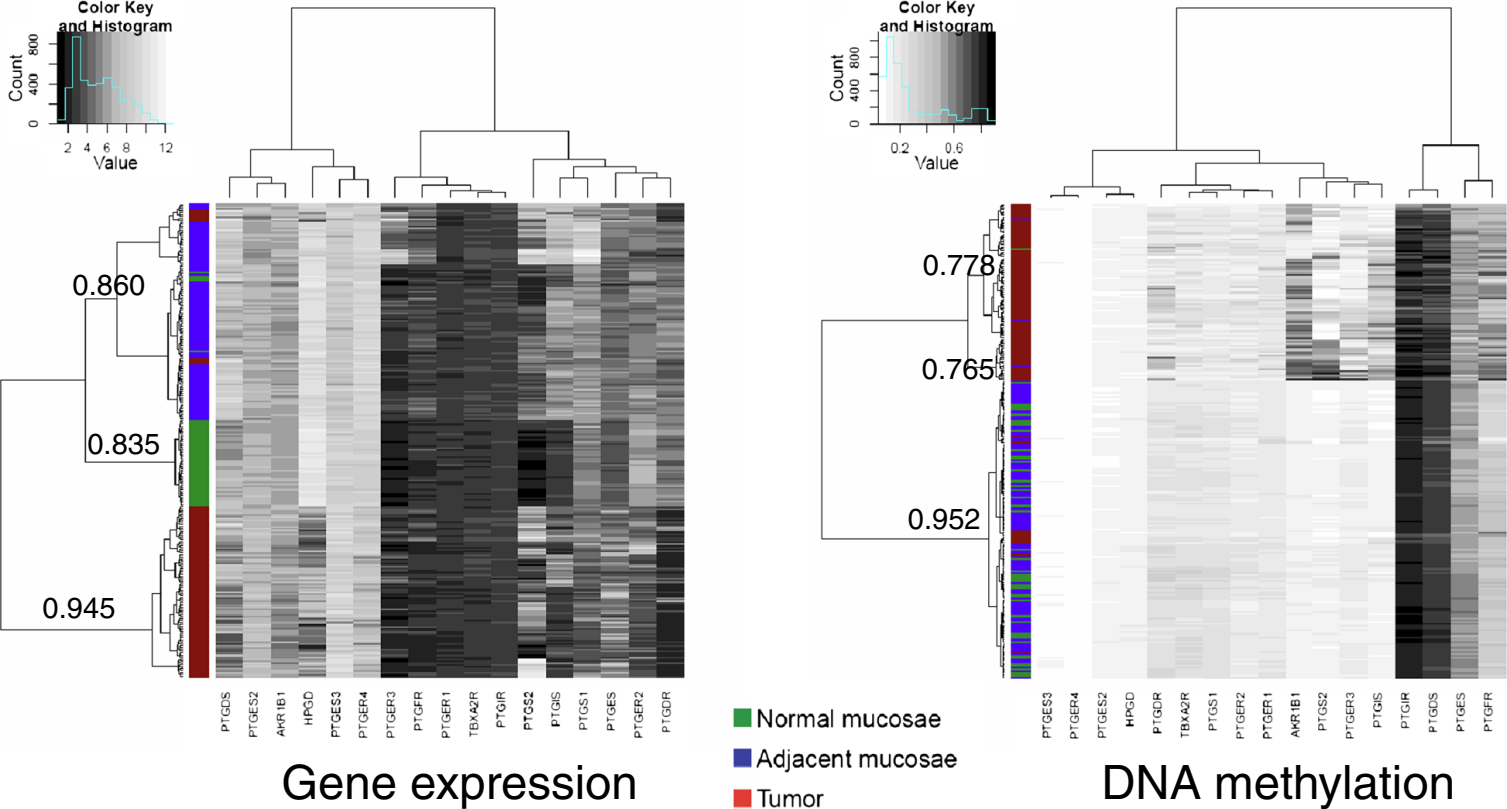
PTGS pathway gene expression and DNA methylation alterations during colorectal tumorigenesis. Hierarchical clustering analysis of gene expression and DNA methylation levels for the 98 CRC patients and 50 healthy donors from the Colonomics dataset. The Jaccard similarity index for sample clustering is indicated next to tree branches. Expression patterns are markedly different not only in tumors (*red*), but also in adjacent mucosae from patients (*blue*), in comparison to healthy donor colonic mucosae (*green*). In contrast, only tumors cluster separately in terms of DNA methylation profiles

Our comparisons of tumors with adjacent mucosae and normal colonic tissue from healthy donors revealed differences in both gene expression and DNA methylation levels. To elucidate if the observed alterations could provide a signature for each type of tissue, we applied unsupervised hierarchical clustering analysis (see Supplementary Methods section in Additional file 1 for more details) to the data obtained for 98 patients and 50 healthy donors. Our analysis revealed that tumors present a different profile for both features, forming a separate cluster in both analyses, whereas adjacent mucosae from patients can only be distinguished from normal mucosae in terms of gene expression, being their DNA methylation profiles indistinguishable (Fig. 3). In all cases, the Jaccard similarity index obtained as a result of the bootstrap resampling was above 0.75, indicating the clusters’ stability.

### Deregulation of the PTGS pathway in colorectal cancer and clinicopathological correlates

As we have shown above, tumors exhibited distinctive signatures of both gene expression and DNA methylation, but some differences were also observed within tissue types. Therefore, we analyzed clinicopathological correlates of gene expression and DNA methylation signatures to determine whether these differences could be associated with features of the patients. Mucosae from control patients did not show any difference regarding age, sex, or location for both gene expression and DNA methylation (Additional file 1: Tables S3 and S4), except for *PTGFR*, that was slightly more methylated in older individuals. This trend was also observed in the adjacent tissue of cancer patients. Strikingly, methylation differences were observed in eight genes between left and right side normal tissues of patients, but only one of them (*PTGDS*) also showed expression differences (hypomethylated and overexpressed in right colon versus left colon) (Additional file 1: Tables S3 and S4). Principal component analysis of DNA methylation data revealed that patients with tumors in the left side of the colon tend to have distinctive methylation profiles, while those with tumors in the right side overlap with both right and left colonic mucosa of controls (Additional file 1: Figure S4).

Tumor molecular profiles showed few associations with clinicopathological variables, and these included downregulation of *PTGDR* and *PTGS1* in older patients, overexpression of *PTGER2*, and hypermethylation of *PTGS2* in right side tumors as compared with left colon (Additional file 1: Tables S3 and S4). Regarding prognosis, we found neither gene expression nor DNA methylation altered signatures as robust predictors of disease-free or overall survival (data not shown). Nevertheless, a quick analysis of gene expression data released in public databases suggests that the expression levels of many genes of the PTGS pathway are associated with poor prognosis (Additional file 1: Figure S5). Availability of follow-up data from large cohorts in the near future should clarify this issue.

## Discussion

In cancer, the most frequent genetic alterations are found in a number of pathways that regulate crucial cellular processes [38, 39]. However, the majority of studies are focused on the role of individual genes, overlooking their biological context. The PTGS metabolic pathway is aberrantly regulated in cancer, being the overexpression of PTGS2 a major feature of many tumor types [6]. Nevertheless, this study and others [33, 40, 41] have shown that only a fraction of CRC present PTGS2 overactivation. This observation together with the fact that PTGS2 targeting is associated with increased cardiovascular risk [24, 25] raises the necessity to develop better strategies to specifically target this signaling pathway before and after tumor appearance.

It is well established that DNA methylation alterations are a frequent feature of many types of cancer, including colorectal cancer [29, 30, 28]. A noteworthy work from Grady and colleagues has demonstrated genome-wide aberrant DNA methylation patterns in early stages of colorectal cancer progression [36]. In particular, there is strong evidence that the PTGS pathway is deregulated at both transcriptional and epigenetic levels in colorectal cancer (reviewed in reference [31]). Nevertheless, the overall landscape of the PTGS pathway in CRC had not been outlined yet. In line with these observations, we have focused this work in the differential DNA methylation of PTGS pathway genes in CRC, having profiled data from a total of 502 tumors. This revealed up to 12 genes that are suppressed in CRC (Fig. 3), including genes that had been previously found misregulated in cancer, such as *HPGD*, *PTGIS*, *PTGER3*, and *AKR1B1* [31].

Even though we analyzed the DNA methylation contents of tumors from four independent collections and applied two different methods, there was remarkable consistency among all datasets. The gene *PTGER1* was the only one showing clear discrepancies, which we believe are due to the different location of the array probes and the oligonucleotides used in dissociation curve analysis.

We have found four genes (*AKR1B1*, *PTGIS*, *PTGER3*, *PTGFR*) hypermethylated in a high proportion of all analyzed tumors (70, 63, 45, and 50 %, respectively), suggesting that DNA methylation is an important mechanism involved in the deregulation of this pathway in CRC. Even though downregulation of *PTGER3* and *AKR1B1* had been previously reported [42–44], this is the first time that their DNA hypermethylation is reported in CRC, whereas the epigenetic silencing of *PTGIS* and *PTGFR* had been previously observed [35, 45]. Importantly, the methylation abnormalities appear to occur early in tumorigenesis, as their frequency was similar in adenomas and carcinomas (Additional file 1: Figure S2). This result, together with the absence of DNA methylation abnormalities in the adjacent normal tissue of CRC patients, suggests that epigenetic alterations may have a critical role in the overriding of the pro-inflammatory status towards a malignant phenotype.

We also interrogated the TCGA database for mutations in these genes, having found little evidence of downregulation of PTGS pathway genes in CRC due to genetic alterations. These results further support DNA methylation as a major gene silencing mechanism involved in this process. Still, other mechanisms cannot be fully discarded, particularly for *AKR1B1* and *PTGFR*, whose expression is not recovered in DNMT-deficient cells.

**Fig. 4:**
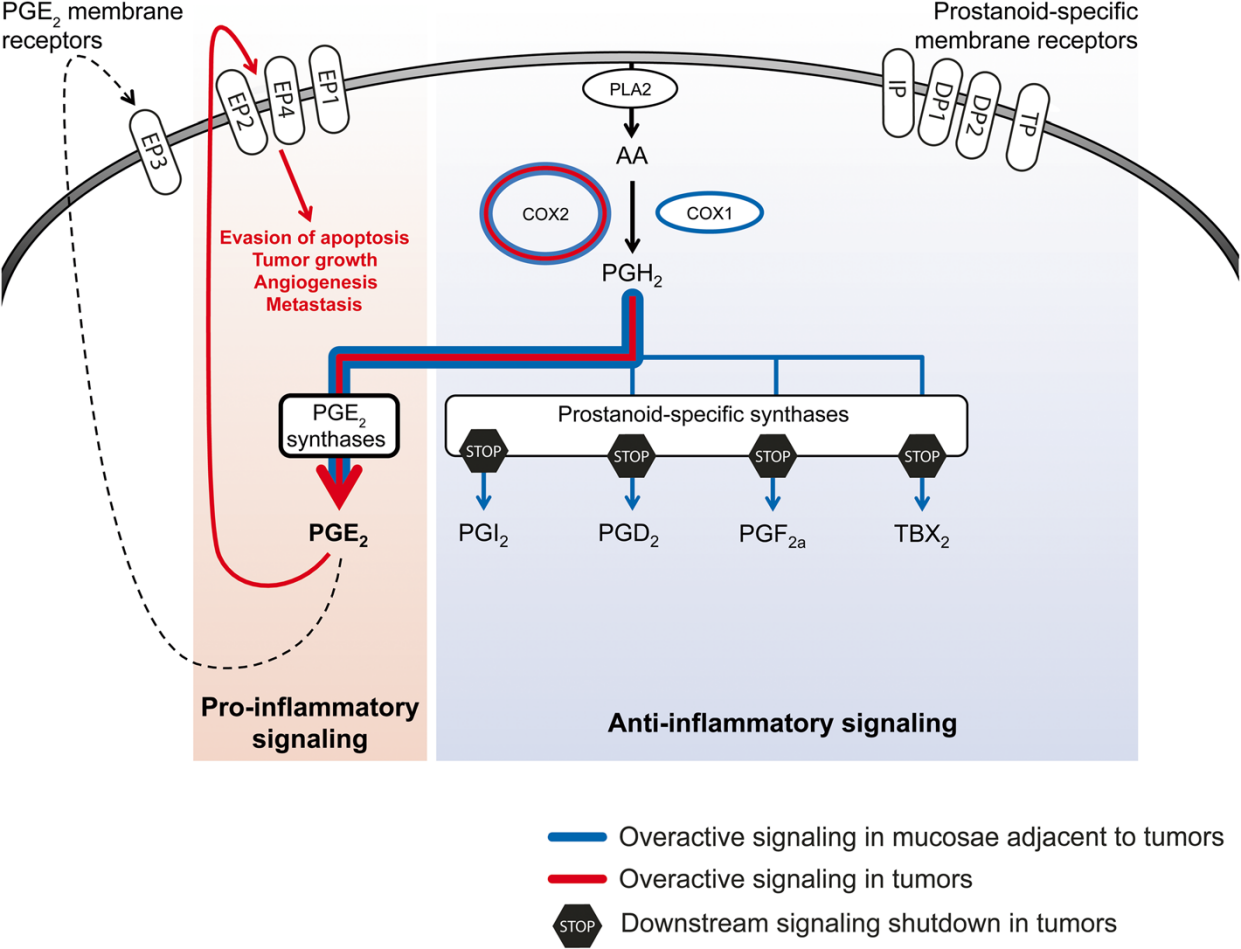
Model for PTGS pathway deregulation during colorectal tumorigenesis. Inflammatory processes, including overactivation of PTGS signaling, are present in the colonic mucosae during, and possibly before, tumor development. In the tumor, promoter hypermethylation and other mechanisms contribute to the repression of several prostanoid-specific synthases and membrane receptors. This leads to the prevalence of PGE_2_ as the major prostanoid in tumor cells, whose downstream actions may include evasion of apoptosis, tumor growth, angiogenesis, and metastasis

Our experimental design included the analysis of colonic mucosae collected from healthy individuals, which allowed us to detect pre-oncogenic alterations already present in the adjacent mucosae of CRC patients. We observed a marked hyperactivation of the pathway at the expression level in adjacent mucosae from patients, which likely reflects a highly inflammatory state of the tissue (Fig. 4). Whether this inflammatory state is cause or consequence of the tumor development remains to be fully understood.

Our results also suggest that the establishment of an inflammatory state in this tissue precedes and possibly contributes to the epigenetic alterations we observe in tumors. In this regard, a connection between PTGS signaling and epigenetic changes has been proposed [46, 47]. Xia and collaborators found compelling evidences supporting a role of PGE_2_ promoting gene silencing by DNA methylation in CRC [47].

Even though our analysis has only been focused on transcriptional and epigenetic profiling of a series of CRC tumors, our data strongly suggests that colorectal cancers present a strong bias towards PGE_2_ production and pro-oncogenic signaling in detriment of the other prostanoids (Fig. 4). The overall deregulation of the pathway results in protection of the PGE_2_ biosynthetic pathway (*PTGES*, *PTGES2*, and *PTGES3*) and strong downregulation of *HPGD*, the responsible for PGE_2_ degradation in the cytosol. This is concordant with PGE_2_ being the major prostanoid in tumor microenvironments [6, 48] and fits previous observations in *Apc*^*Min*^ transgenic mice, where deletion of PTGER1, PTGER2, and PTGER4 inhibits the development of CRC [49–51]. Interestingly, each one of the PGE_2_ receptors displayed a distinctive behavior. PTGER3 and PTGER4 were upregulated in adjacent mucosa, which could indicate the permissiveness of the host tissue to PTGS signaling. Both receptors appear to have similar roles in stromal regulation [52], but show different behaviors in the tumor: PTGER3 is the only receptor whose overexpression is maintained in the tumor tissue (Fig. 1b). This could be related with its reported role in increasing tumor growth, as opposed to the rest of the receptors [53]. PTGER2 showed consistent downregulation in both normal and tumor tissue.

A recent study has shown that in vivo administration of celecoxib (an inhibitor of PTGS2) abrogates chemoresistance in xenografted tumors derived from a patient who was resistant to chemotherapy. These results strongly suggest the participation of the PTGS/PGE_2_ signaling axis in the reactivation of cancer stem cells, a major cause of treatment failure [17].

Our integrative approach has contributed to explore the intricacies of the PTGS pathway in colorectal cancer, revealing a dual disruption: the adjacent non-tumor tissue of colorectal cancer patients shows global transcriptional upregulation of the PTGS pathway; whereas, cancer cells restrict the hyperactivation towards PGE_2_ signaling, which is achieved by epigenetic silencing of competing eicosanoid synthases and receptors, even in early stages of tumor progression. The possible involvement of epigenetic alterations in malignant cell transformation within a pro-inflammatory microenvironment releases new candidate biomarkers for prevention and early diagnosis, as well as potential new therapeutic targets. Taking into account the likely contribution of this pathway to the etiology and progression of the disease, further functional studies are required to understand the mechanisms underlying PTGS regulation and signaling in both normal and malignant cells. The ability to modulate this pathway may constitute a powerful tool to prevent and eventually treat colorectal cancer.

Finally, the influence of environmental factors cannot be excluded from the etiology of CRC. As a metabolic pathway, the PTGS pathway signaling is a result of an individual’s metabolic state, being directly responsive to environmental exposures. On the other hand, diet and lifestyle factors are also known to contribute to aberrant epigenetic signatures in colorectal cancer (reviewed in [54–56]). In an omics era, patients’ clinical pictures and response to therapy should be seen as the result of their unique genome, epigenome, transcriptome, proteome, metabolome, as well as of their interactome and exposome [56, 57]). The emerging concept of molecular pathological epidemiology (MPE) [57], which aims to integrate all contributing factors in one discipline, should bring new queues of personalized medicine to CRC patients. Future studies should aim to go further and integrate studies like ours with other layers of information such as diet in order to better prevent and treat cancer patients.

## Conclusions

This study shows that the PTGS signaling pathway displays a pro-inflammatory molecular signature in the non-tumor colonic mucosa of colorectal cancer patients. Noteworthy, in colorectal adenomas and carcinomas, epigenetic mechanisms (namely DNA hypermethylation) redirect the transcriptional deregulation of the non-tumor environment towards selective activation of PGE_2_ function and blockade of other biologically active prostaglandins. These results have important implications for the proper design and application of preventive and therapeutic strategies targeting prostaglandin metabolism. The large arsenal of available agonists and antagonists of prostaglandins demand an individualized analysis of the PTGS pathway not only in patients, but also in individuals at risk of developing CRC.

## Availability of supporting data

All sequencing and array data supporting the results of this article are available through Gene Expression Omnibus with accession numbers GSE44076 and GSE48684, and the TCGA data through cBioportal (www.cbioportal.org).

## Methods

### Patients

Four series of colorectal cancer patients were used in this study (Additional file 1: Table S1). The first series included a total of 64 colorectal cancer patients from the Institut Català d’Oncologia (L’Hospitalet, Barcelona, Spain) and Hospital Germans Trias i Pujol (Badalona, Barcelona, Spain). Gene expression and DNA methylation analyses were performed in tumor and adjacent normal mucosae as described below. The most important clinicopathological information of patients and healthy controls is presented in Additional file 1: Table S5. The study was approved by the Hospital Germans Trias i Pujol ethical committee. The individuals gave their written informed consent.

Data from the *Colonomics* project (www.colonomics.org) was used as second series and comprised 98 colon tumors and paired pathologically adjacent normal mucosa samples (minimum distance of 10 cm from the tumor). Pathologists confirmed all colon cancer diagnoses and selected fresh tissue samples from tumor and normal mucosa taken from the proximal resection margin. Fifty tissue samples of non-cancer colon mucosa, with no adenomas and no family cancer history reported, were obtained through colonoscopy. The most important clinicopathological information of patients and healthy donors is presented in Table 1.

Data for a third cohort of 254 colorectal tumors, 38 of which with adjacent tissues, were obtained from The Cancer Genome Atlas (TCGA) (www.cancergenome.nih.gov). DNA methylation data of other additional 15 cancers types were also extracted from the TCGA (see Additional file 1). Finally, DNA methylation data recently generated by Luo et al. [36] using the HumanMethylation450K array in 19 colon mucosae from healthy individuals, 22 adjacent non-tumor tissues, 42 adenomas, and 64 carcinomas were also included.

Cell lines used in the study are described in Additional file 1.

### Gene expression analysis

For the preliminary analysis, total RNA was extracted from tissues and cell lines and quantified by real-time PCR, applying the delta Ct method (primers are listed in Additional file 1: Table S6). Expression analysis of the *Colonomics* series was performed as described [58]. Both raw and normalized data are available in the Gene Expression Omnibus (GEO) database with accession number GSE44076.

### DNA methylation analysis

Genomic DNA of five cell lines, tumors, and adjacent mucosae from 64 patients was treated with the EZ DNA Methylation™ Kit (Zymo Research) to evaluate their methylation status by bisulfite sequencing and dissociation curve analysis as described [59] (primers are listed in Additional file 1: Table S7). Samples were analyzed in triplicates.

Methylation data in the *Colonomics* series was collected with Infinium HumanMethylation450 array. Further details are available as Additional file 1.

### Statistical analysis

ANOVA was applied to determine the significant differences between three groups of samples (normal mucosae, adjacent tissue, and CRC tumors) for both gene expression and DNA methylation. Genes with statistically significant differences (*P* < 0.05) were further evaluated using the Tukey’s HSD (honest significant difference) test (Table 2).

Wilcoxon test was applied to set differences between clinical variables and gene expression and DNA methylation of Colonomics data. Adjusted *p* value was obtained using Benjamin and Hochberg method (Additional file 1: Table S3 and S4).

Free and overall survival analysis was performed for both gene expression and DNA methylation data using tertiles. A log-rank univariate test was performed for each gene and a Kaplan-Meier curve was built. Only genes with a *P* value <0.2 in the univariate analysis were considered for the multivariate analysis. Finally, with these genes, a Cox Proportional Hazards model was built.

Hierarchical clustering was performed using the Ward method with euclidean distances. We set the number of clusters to 3 to see how well the three types of tissues are classified. The Jaccard similarity index was obtained by bootstrap resampling to assess the stability of the clusters. A valid, stable cluster should yield a mean Jaccard similarity value of 0.75 or more.

## Additional file

Additional file 1:
**Supplementary information.** Supplementary methods, supplementary references, Tables S1–S7, and Figures S1–S5.

## Abbreviations

CRC: colorectal cancer
TCGA: The Cancer Genome Atlas
