# Supplementary material for: Epigenetics override pro-inflammatory PTGS transcriptomic signature towards selective hyperactivation of PGE2 in colorectal cancer
Source: Clin Epigenetics. 2015 Jul 24;7(1):74. doi: 10.1186/s13148-015-0110-4 (PMC4512023; doi:10.1186/s13148-015-0110-4)
Supplement: Additional file 1: — Supplementary information. Supplementary methods, supplementary references, Tables S1–S7, and Figures S1–S5. [file 13148_2015_110_MOESM1_ESM.pdf]

Supplementary Information for

Epigenetics override proinflammatory PTGS transcriptomic signature towards selective hyperactivation of PGE_2 in colorectal cancer

Clinical Epigenetics, 2015

Inês Cebola, Joaquin Custodio, Mar Muñoz, Anna Díez-Villanueva, Laia Paré, Patricia Prieto, Susanna Aussó, Llorenç Coll-Mulet, Lisardo Boscá, Victor Moreno, Miguel A. Peinado*

*Correspondence to: Miguel A. Peinado. e-mail: map@imppc.org

Additional File 1:

Supplementary Methods	Pages 2-3
Supplementary references	Page 4
Tables S1-S7	Pages 5-14
Figures S1-S6	Pages 15-19

SUPPLEMENTARY METHODS

TCGA data

Methylation data of 16 cancers types (COAD, STAD, READ, PAAD, THCA, BLCA, BRCA, LGG, PRAD, KIRC, KIRP, LUSC, GBM, LUAD, UCEC, LIHC) were downloaded from The Cancer Genome Atlas (TCGA) (www.cancergenome.nih.gov). For each tumor type, paired tumors and adjacent normal mucosae were analyzed, together with additional unmatched tumors. Sample preparation details can be found in the TCGA documentation section. For our analysis, we used the beta-values obtained from the level_3 methylation data. Samples were considered hypermethylated when their mean beta-value was at least the double of the mean of the controls (considering all probes in the area).

Cell lines

Cell lines HCT116, HT-29, DLD-1, SW480, LoVo were obtained from the American Type Culture Collection (ATCC) and grown in Dulbecco's Modified Eagle Medium (DMEM)/F12 (1:1) supplemented with 100mM Sodium Pyruvate, 200mM L-Glutamine, 10% FBS (Invitrogen), at 37ºC in humidified 95% air and 5% CO_2. HCT116-DKO cells (HCT116 deficient in both DNA methyltransferases [1]) were cultured under the same conditions.

Methylation data

Genomic DNA was extracted from peripheral leukocytes by the salting-out procedure (Miller et al, 1988) QIAmp. The extracted DNA was quantified using a Nano Drop ND 2000c spectrophotometer (NanoDrop Thermo scientific, Wilmington, DE) and stored at 4C. 200-600 ng of DNA were treated with bisulfite using EZ DNA methylation kit (Zymo Research. Cat. No. D5004) according to manufacturer's recommendations. The incubation profile was 16 cycles at 95C for 30s, 50C for 60 min and a final holding step at 4C [2].
DNA methylation profiles were generated using the Illumina Human Methylation 450K BeadChip assay (technical details of this array are described elsewhere [3]). This array involves whole genome amplification, interrogate the methylation levels of 485,577 CpG sites on bisulfite-treated DNA, covering 99% of RefSeq genes and 96% of CpG island regions such as CpG island, island shore and shelf, 5' and 3' UTRs, and promoter and gene body [3]. The methylation level is represented by the β value resulting from the ratio of intensity signal obtained from the methylated allele over the sum of methylated and unmethylated allele intensity bead signals. β values may take any value between 0 (non-methylated) and 1 (completely methylated).
We used raw *.idat files as a starting point for this pipeline, based on the Bioconductor library "minfi". Here, the outliers were removed by inspecting the overall signal intensity, the distribution of M-values and the control probe profile. We also checked on the sex specification obtained from the results of the Affymetrix Genome-Wide Human SNP 6.0 array (Affymetrix, Santa Clara, USA), using the observed genotypes of SNPs on chromosome X and Y. All individuals who were marked as "male" but with significant amount of heterozygous X genotypes (>=1%), or who were marked as "female" but with high frequency of homozygous X genotypes (>=80%) or Y genotype readings, were individually inspected against original data source. If no satisfactory correction could be obtained these individuals were excluded from further analyses. A clustering between the 19 common SNPs of the HumanMethylation450K array and affy 6.0 to check the correct pairing between N-T was performed. A total of 17 samples were excluded for these reasons. Thus, a final dataset of 240 arrays was used for subsequent analyses.
Probes with a minfi detection p value > 0.01 exceeding 5% of the samples were annotated as missing, we removed probes on chromosomes X and Y and the assays measuring 65 SNPs (from Illumina manifest). Afterwards, subset-quantile within array normalization (SWAN) introduced sub-quantile

normalization for the methylated and unmethylated channels separately, assuming that the distribution of the probes with similar number of CpGs should be similar, irrespective of probe type (n=481,019) [4]. Methylation probes with low variability were removed (SD < 0.04). We discarded 41,082 probes that ambiguously mapped to multiple locations in the human genome with up to two mismatches [5]. We excluded a further 11,765 probes that contained SNPs within 10 bp of the CpG interrogate (from 1,000 genomes project, CEU population). This resulted in a final set of 430,158 probes that were used in all further analyses. We mapped the 485,577 Illumina probes to the human genome sequence (hg18) using UCSC genome browser having a complete annotation of all genes.
To set the methylation beta value of each gene promoter we used the median of the beta values of probes located at the 5'UTR, and within 1.5 Kb of the TSS according to the UCSC genome browser annotation. Probes are listed below:

**Gene (HumanMethylation450K array probes)**
**PTGES2** (cg01311102 cg13880225 cg14092536 cg22863798 cg14324338 cg10218959 cg13826862 cg13833419 cg16020551 cg05930684 cg24730207)
**PTGS2** (cg18335243 cg08482694 cg17419623 cg09461185 cg24887140 cg26564040 cg10180406 cg25837803 cg23070111 cg25147026 cg16101346)
**PTGES3** (cg04686114 cg12507325 cg03226218 cg16823406 cg08027110 cg20253639 cg07281688 cg11439834 cg22366350)
**PTGES** (cg04831490 cg17683775 cg14390764)
**TBXA2R** (cg06384607 cg25307468 cg15861540 cg04985611 cg09998229 cg08069088 cg11729169 cg18738857 cg07481491 cg22757101 cg19225308 cg01209635 cg23846712 cg05343404)
**PTGER2** (cg21502048 cg16134491 cg26674715 cg06738602 cg20055841 cg08423052 cg23693907)
AKR1B1 (cg18416881 cg16132520 cg13801416 cg06864853 cg10795359 cg14629509 cg21079345 cg02215070 cg09957386 cg08167706 cg23918923 cg04663564)
**PTGER1** (cg10468702 cg21533997 cg24360651 cg03949996 cg27524460 cg03020379
**PTGIR** cg03311682 cg11822964 cg07844738 cg04706667)
**PTGDR** (cg18369034 cg23022053 cg10837806 cg24089118 cg18242103 cg24989962 cg17929687 cg02191312 cg05302386 cg09516965)
**PTGER4** (cg06802812 cg08164315 cg06013215 cg03337243 cg17421097 cg02026948 cg04727116 cg21149775 cg25664622 cg01952088 cg01897756 cg04126707 cg04790357 cg04824304 cg26597539)
**PTGDS** (cg00563932 cg18502630)
**PTGER3** (cg23647191 cg14963735 cg12739034 cg03726881 cg00915178 cg20785136 cg00781169)
**PTGIS** (cg11746287 cg15543919 cg16537756)
**PTGFR** (cg04410756 cg09685982 cg24022301 cg03949391 cg27046936 cg03495868 cg24924936 cg00701741 cg02859837 cg19403909)
**PTGS1** (cg13974165 cg00501774 cg10493166 cg14154487 cg12927153 cg13630095)
**HPGD** (cg03772063 cg06366981 cg02822257 cg07372795 cg11073923 cg00906130 cg18164599 cg15474754 cg13181537 cg05527430 cg04555941 cg01084566 cg20455617)

SUPPLEMENTARY REFERENCES

1. Rhee I, Bachman KE, Park BH, Jair KW, Yen RW, Schuebel KE et al. DNMT1 and DNMT3b cooperate to silence genes in human cancer cells. Nature. 2002;416:552-6.

2. Sandoval J, Heyn H, Moran S, Serra-Musach J, Pujana MA, Bibikova M et al. Validation of a DNA methylation microarray for 450,000 CpG sites in the human genome. Epigenetics. 2011;6:692-702.

3. Bibikova M, Barnes B, Tsan C, Ho V, Klotzle B, Le JM et al. High density DNA methylation array with single CpG site resolution. Genomics. 2011;98:288-95.

4. Maksimovic J, Gordon L, Oshlack A. SWAN: Subset-quantile within array normalization for illumina infinium HumanMethylation450 BeadChips. Genome Biol. 2012;13:R44.

5. Price ME, Cotton AM, Lam LL, Farre P, Emberly E, Brown CJ et al. Additional annotation enhances potential for biologically-relevant analysis of the Illumina Infinium HumanMethylation450 BeadChip array. Epigenetics Chromatin. 2013;6:4.

6. Weinstein JN, Collisson EA, Mills GB, Shaw KR, Ozenberger BA, Ellrott K et al. The Cancer Genome Atlas Pan-Cancer analysis project. Nat Genet. 2013;45:1113-20.

7. Luo Y, Wong CJ, Kaz AM, Dzieciatkowski S, Carter KT, Morris SM et al. Differences in DNA methylation signatures reveal multiple pathways of progression from adenoma to colorectal cancer. Gastroenterology. 2014;147:418-29 e8.

8. Frigola J, Muñoz M, Clark SJ, Moreno V, Capella G, Peinado MA. Hypermethylation of the prostacyclin synthase (PTGIS) promoter is a frequent event in colorectal cancer and associated with aneuploidy. Oncogene. 2005;24:7320-6.

9. Kamphuis W, Schneemann A, van Beek LM, Smit AB, Hoyng PF, Koya E. Prostanoid receptor gene expression profile in human trabecular meshwork: a quantitative real-time PCR approach. Invest Ophthalmol Vis Sci. 2001;42:3209-15.

10. Mizuno H, Kitada K, Nakai K, Sarai A. PrognoScan: a new database for meta-analysis of the prognostic value of genes. BMC Med Genomics. 2009;2:18.

Table S1. Samples used in this study

Series	Tissue Type	no of samples	Molecular data	Data access	Reference
HGTiP/ICO	tumor and paired adjacent normal	64 cancers<br>64 adjacent	Gene expression, DNA methylation	this paper	this paper
Colonomics	Healthy mucosa, tumor and adjacent normal	50 healthy<br>98 cancers<br>98 adjacent	Gene expression, DNA methylation	http://www.colonomics.org	this paper
TCGA	tumor and adjacent normal	254 cancers<br>38 adjacent	DNA methylation	https://tcga-data.nci.nih.gov/tcga/	Weinstein et al [6]
Luo et al.	Healthy mucosa, tumor and adjacent normal	19 healthy<br>42 adenomas<br>22 adjacent<br>64 cancers	DNA methylation	GEO: GSE48684	Luo et al [7]

Table S2. Nomenclature of the PTGS pathway genes analyzed in this study.

Gene	Protein	Alternative name
AKR1B1	Aldo-keto reductase family 1, member B1	PGFS (prostaglandin F2alpha synthase)
HPGD	Hydroxyprostaglandin dehydrogenase 15-(NAD)	
PTGDR	Prostaglandin D2 receptor	DP
PTGDS	Prostaglandin D2 synthase	
PTGER1	Prostaglandin E2 receptor 1	EP1
PTGER2	Prostaglandin E2 receptor 2	EP2
PTGER3	Prostaglandin E2 receptor 3	EP3
PTGER4	Prostaglandin E2 receptor 4	EP4
PTGES	Prostaglandin E2 synthase	
PTGES2	Prostaglandin E2 synthase 2	
PTGES3	Prostaglandin E2 synthase 3 (cytosolic)	cPGES
PTGFR	Prostaglandin F2alpha receptor	FP
PTGIR	Prostacyclin receptor	IP
PTGIS	Prostacyclin synthase	PGIS
PTGS1	Prostaglandin-endoperoxide synthase 1	COX-1 (Cyclooxygenase-1)
PTGS2	Prostaglandin-endoperoxide synthase 2	COX-2 (Cyclooxygenase-1)
TBXA2R	Thromboxane A2 receptor	TP

Table S3. Clinicopathological correlates of gene expression in the mucosa of healthy controls (NORMAL) and in CRC patients (ADJACENT MUCOSA and TUMOR)*.

	NORMAL MUCOSA			ADJACENT MUCOSA (AM)					TUMOR (T)					FOLD CHANGE (LOG2(T/AM))				
	Sex	Age	Site	Stage	Sex	Age	Location	Metastasis	Stage	Sex	Age	Site	Metastasis	Stage	Sex	Age	Site	Metastasis
PTGES2	0.4178	0.5689	0.9161	0.8712	0.5026	0.9031	0.9853	0.9289	0.7230	0.3529	0.4890	0.3072	0.8820	0.8061	0.7458	0.7956	0.4485	0.7412
PTGS2	0.8169	0.7871	0.0660	0.5679	0.3495	0.3174	0.9853	0.9289	0.7985	0.3529	0.4105	0.8277	0.8820	0.8061	0.9810	0.1546	0.9458	0.8752
PTGES3	0.5617	0.5689	0.3823	0.8712	0.8988	0.1724	0.8277	1.0000	0.7230	0.7501	0.7930	0.7585	0.8820	0.8061	0.9810	0.5410	0.5458	0.8752
PTGES	0.5617	0.7871	0.3790	0.6451	0.5761	0.2311	0.2800	0.9289	0.7333	0.3529	0.7959	0.9119	0.8820	0.8061	0.9097	0.7956	0.9458	0.8752
TBXA2R	0.4882	0.7295	0.9385	0.6451	0.5761	0.9031	0.9855	0.9289	0.7230	0.4632	0.4890	0.8277	0.8820	0.8061	0.6911	0.7956	0.9485	0.7412
PTGER2	0.4178	0.5689	0.0660	0.5679	0.8988	0.3284	0.1304	0.9289	0.8956	0.7808	0.7959	0.0192	0.8820	0.8508	0.9779	0.8311	0.2080	0.8752
AKR1B1	0.6053	0.5689	0.3990	0.5679	0.8571	0.9031	0.8277	0.9289	0.9431	0.3529	0.7959	0.6058	0.8820	0.8061	0.7458	0.7956	0.9458	0.8752
PTGER1	0.4882	0.7871	0.2673	0.6793	0.5761	0.9583	0.8277	1.0000	0.8523	0.6061	0.3805	0.9119	0.8820	0.8061	0.6911	0.6756	0.9485	0.8752
PTGIR	0.7894	0.7871	0.4982	0.8712	0.8653	0.9031	0.4130	0.9289	0.7230	0.3529	0.8753	0.6037	0.8820	0.8061	0.6911	0.8311	0.3156	1.0000
PTGDR	0.6053	0.5689	0.3790	0.8712	0.3495	0.9031	0.4130	0.6834	0.7230	0.5106	0.0408	0.3072	0.8820	0.8061	0.9810	0.2891	0.2080	0.8752
PTGER4	0.4882	0.7871	0.4539	0.6238	0.5761	0.9031	0.5524	1.0000	0.9256	0.7808	0.7959	0.6037	0.8820	0.8061	0.9440	0.7956	0.3156	0.8752
PTGDS	0.4178	0.7295	0.2673	0.6451	0.8988	0.3174	0.0288	1.0000	0.7230	0.5106	0.8753	0.6037	0.8820	0.8061	0.7458	0.7956	0.9458	0.8752
PTGER3	0.7150	0.7670	0.3790	0.6451	0.8653	0.7221	0.8277	0.9289	0.7985	0.3529	0.7930	0.9855	0.8820	0.8506	0.7458	0.8311	0.9458	0.8752
PTGIS	0.5617	0.7871	0.3790	0.5679	0.5026	0.9031	0.9663	0.9289	0.7230	0.3529	0.6108	0.8523	0.8820	0.8061	0.9779	0.6756	1.0000	0.8752
PTGFR	0.4882	0.7871	0.1110	0.6451	0.8988	0.9031	0.5524	0.6834	0.8523	0.3529	0.7673	0.6037	0.8820	0.8061	0.6911	0.7956	0.9458	0.7412
PTGS1	0.1321	0.7670	0.1268	0.5679	0.3495	0.9031	0.8277	0.9289	0.7230	0.5106	0.0408	0.6037	0.8820	0.8508	0.9779	0.1546	0.9458	0.8752
HPGD	0.7894	0.5689	0.3790	0.8712	0.8988	0.9031	0.4130	0.8610	0.7985	0.3529	0.7959	0.3011	0.8820	0.8061	0.6911	0.7956	0.2080	0.8752

* Gene expression was analysed with *SurePrint* arrays. Data were obtained from the *Colonomics* project and show Wilcoxon P values adjusted using Benjamin and Hochberg method.

Gene expression values in regard to the features that are statistically significant (from Table S3)

NORMAL

LOCATION		PTGDS		
		MEAN	SD	P
site_Left (n=61)		7.4264	0.9349	0.0288
site_Right (n=39)		8.0669	1.1159	

TUMOR

AGE	PTGDR		
	MEAN	SD	P
age_<=65 (n=26)	3.3739	1.4867	0.0408
age_>65 (n=74)	2.5285	0.6461	

AGE	PTGS1		
	MEAN	SD	P
age_<=65 (n=26)	4.9576	1.0601	0.0408
age_>65 (n=74)	4.2757	0.9525	

LOCATION	PTGER2		
	MEAN	SD	P
site_Left (n=61)	3.8527	0.8970	0.0192
site_Right (n=39)	4.8345	1.5711	

Table S4. Clinicopathological correlates of DNA methylation in the mucosa of healthy controls (NORMAL) and in CRC patients (ADJACENT MUCOSA and TUMOR)*.

	NORMAL MUCOSA			ADJACENT MUCOSA (AM)					TUMOR (T)					FOLD CHANGE (LOG2(T/AM))				
	Sex	Age	Site	Stage	Sex	Age	Site	Metastasis	Stage	Sex	Age	Site	Metastasis	Stage	Sex	Age	Site	Metastasis
PTGES2	0.9670	0.9745	0.5272	0.8843	0.5233	0.7111	1.0000	0.9229	0.6120	0.6366	0.6029	0.9164	0.4045	0.2976	0.9398	0.9712	0.7733	0.6260
PTGS2	0.7344	0.3879	0.4412	0.7105	0.5233	0.9648	0.0003	0.9229	0.8218	0.9638	0.4661	0.0045	0.9996	0.4973	0.6197	0.8025	0.0776	0.7049
PTGES3	0.7344	0.9745	0.4412	0.8843	0.8580	0.9648	0.3016	0.9229	0.3513	0.9638	0.1518	0.8415	0.9996	0.9668	0.9398	0.5515	0.6336	0.6993
PTGES	0.7344	0.9745	0.7467	0.7105	0.3636	0.3876	0.0953	0.9229	0.6120	0.9638	0.2437	0.6323	0.9996	0.9061	0.6239	0.8676	0.7733	0.6993
TBXA2R	0.7344	0.9745	0.7467	0.9155	0.5233	0.7111	0.0057	0.9996	0.3513	0.9638	0.1518	0.3276	0.4045	0.2812	0.9398	0.0618	0.9840	0.6260
PTGER2	0.9662	0.8878	0.4412	0.8843	0.4070	0.9648	0.0319	0.9229	0.3513	0.9638	0.1518	0.9164	0.3843	0.2106	0.9398	0.3336	0.7733	0.6993
AKR1B1	0.6770	0.9745	0.1671	0.9155	0.8369	0.9648	1.0000	0.9996	0.8929	0.2988	0.9795	0.4383	0.9996	0.9162	0.6197	0.9712	0.4973	0.7122
PTGER1	0.7344	0.9745	0.9477	0.8843	0.5233	0.9648	0.0497	0.9229	0.3513	0.2988	0.9795	0.8415	0.9996	0.2812	0.6197	0.9330	0.4973	0.6993
PTGIR	0.8839	0.3087	0.1412	0.8843	0.9114	0.9648	0.0120	0.9229	0.9683	0.9638	0.9748	0.6323	0.9996	0.7744	0.9398	0.9350	0.2995	1.0000
PTGDR	0.3274	0.9917	0.4412	0.8843	0.5839	0.9648	0.0139	0.9229	0.3513	0.9638	0.5581	0.9164	0.4045	0.2812	0.9398	0.8025	0.7733	0.6260
PTGER4	0.7042	0.9745	0.4412	0.8843	0.5233	0.7111	0.5555	0.9229	0.8218	0.9638	0.9748	0.9549	1.0000	0.2812	0.6239	0.8025	0.7733	0.9950
PTGDS	0.4550	0.3087	0.3832	0.9155	0.5233	0.9648	0.0003	0.9229	0.9683	0.9638	0.8915	0.9164	0.9996	0.9162	0.9398	0.9712	0.4973	0.9981
PTGER3	0.7029	0.9745	0.7467	0.8843	0.5233	0.9671	0.0806	1.0000	0.9683	0.9638	0.9748	0.0578	0.9996	0.9668	0.9398	0.9330	0.1363	0.7049
PTGIS	0.3274	0.3136	0.3832	0.8843	0.5064	0.3792	0.5498	0.9229	0.6120	0.9638	0.2437	0.6323	0.9996	0.4973	0.9398	0.5515	0.7733	0.9950
PTGFR	0.7042	0.0129	0.0841	0.9155	0.3636	0.0038	0.7648	0.9229	0.6120	0.9638	0.6169	0.9164	0.9996	0.2976	0.6197	0.8025	0.7733	0.6993
PTGS1	0.7042	0.9745	0.9477	0.8843	0.3636	0.9648	0.0008	0.9229	0.3513	0.9638	0.1745	0.8415	0.5430	0.2812	0.9398	0.3336	0.4973	0.6993
HPGD	0.7042	0.9745	0.9477	0.8843	0.5233	0.9648	0.1179	0.9229	0.3513	0.9638	0.9748	0.8415	0.9996	0.2106	0.6197	0.9712	0.7733	0.9981

* Gene expression was analysed with *SurePrint* arrays. Data were obtained from the *Colonomics* project and Wilcoxon P values adjusted using Benjamin and Hochberg method..

Gene expression values in regard to the features that are statistically significant (from Table S4)

NORMAL MUCOSA

AGE	PTGFR		
	MEAN	SD	P
age_<=65 (n=35)	0.3431	0.0379	0.0129
age_>65 (n=26)	0.3940	0.0528	

ADJACENT MUCOSA

AGE	PTGFR		
	MEAN	SD	P
age_<=65 (n=26)	0.3526	0.0772	0.0038
age_>65 (n=74)	0.3830	0.0575	

LOCATION	PTGS2		
	MEAN	SD	P
site_Left (n=27)	0.1228	0.0791	0.0003
site_Right (n=34)	0.1355	0.0300	
	TBXA2R		
	MEAN	SD	P
site_Left (n=27)	0.2244	0.0396	0.0057
site_Right (n=34)	0.2476	0.0329	
	PTGER2		
	MEAN	SD	P
site_Left (n=27)	0.1770	0.0287	0.0319
site_Right (n=34)	0.1953	0.0306	
	PTGER1		
	MEAN	SD	P
site_Left (n=27)	0.1904	0.0251	0.0497
site_Right (n=34)	0.1969	0.0161	

TUMOR

LOCATION	PTGS2		
	MEAN	SD	P
site_Left (n=61)	0.2091	0.1670	0.0045
site_Right (n=39)	0.3735	0.2304	NA

Continued in next page

ADJACENT MUCOSA

LOCATION	PTGIR		
	MEAN	SD	P
site_Left (n=27)	0.8312	0.0264	0.0120
site_Right (n=34)	0.8208	0.0169	NA
	PTGDR		
	MEAN	SD	P
site_Left (n=27)	0.2255	0.0505	0.0139
site_Right (n=34)	0.2432	0.0342	
	PTGDS		
	MEAN	SD	P
site_Left (n=27)	0.7668	0.0216	0.0003
site_Right (n=34)	0.7473	0.0214	
	PTGS1		
	MEAN	SD	P
site_Left (n=27)	0.2117	0.0269	0.0008
site_Right (n=34)	0.2310	0.0213	

Table S5. Information of CRC patients studied in the expression preliminary analysis and in the DNA methylation dissociation curve analysis.

Id	AGE	SEX	STAGE
CR10	49	M	II
CR12	65	M	III
CR13	86	F	III
CR15	69	M	III
CR16	56	F	III
CR17	76	F	III
CR18	75	F	III
CR19	72	F	III
CR20	63	M	III
BH001	76	M	III
BH002	74	F	II
BH003	84	M	III
BH005	71	M	II
BH006	59	F	III
BH007	48	M	III
BH017	74	F	II
BH021	62	F	III
BH022	84	F	III
BH026	80	F	II
BH027	64	M	III
BH028	68	M	III
BH038	81	M	III
BH046	67	M	II
BH050	73	M	II
BH051	51	F	II
ID009	67	M	II
ID014	64	F	II
ID016	70	F	III
ID017	31	F	III
ID025	67	M	II
ID046	68	M	II
ID063	74	F	III
ID072	73	M	III

Id	AGE	SEX	STAGE
ID078	62	M	II
ID102	78	M	II
ID114	76	F	II
ID118	51	F	III
ID138	80	F	II
ID143	na	F	III
ID145	73	F	II
ID146	65	F	II
ID154	54	M	III
ID186	56	M	II
ID223	75	M	II
ID303	67	F	II
ID346	64	M	II
ID369	76	M	III
ID377	81	M	III
ID385	64	F	III
ID405	na	M	na
ID437	59	F	II
ID450	56	M	III
ID495	71	F	II
ID528	63	F	II
ID541	72	M	II
ID544	61	F	III
ID549	79	M	III
ID551	58	F	III
ID555	49	F	III
ID557	75	M	III
ID563	na	M	na
ID597	na	F	na
ID608	73	F	III
ID628	na	F	na

Table S6. Primers used in this study for the expression analysis of PTGS pathway genes.

Gene	Product position[1]	Forward primer	Reverse primers	Product size (bp)
AKR1B1	chr7:134136426-134143806	CCGTCTCCTGCTCAACAAC	TACACATGGGCACAGTCGAT	138
PTGS1	chr9:125152594-125154599	AAACCCTACACCTCCTTCCA	CCCCAATCTCTATCATACTCT	163
PTGS2	chr1:186643812-186645076	GAATTACCCAGTTTGTTGAATC	TTCTACCAGAAGGGCAGGAT	278
HPGD	chr4:175413148-175414389	AATCAATTGAAAAAGAAGAAAA	GAAAATGAATTCCCTTAGAA	186
PTGDR	chr14:52735343+52741617	ACCGTGCTCTTCACTATGTG	AAATCTTGTGAAAAAATATCCG	205
PTGDS	chr9:139875286+13987611	TAAGTGCATGACGGAACAATA	AGGGGCTCGGGGAAGGAA	110
PTGER1	chr19:14583514-14584209	CTGCTGGAGCCCAATGC	TGGCGCAGTAGGATGTAC	144
PTGER2	chr14:52782009+52794013	GCAGGAGAGGGGAAAGG	AATTGATAAAAACCTAAGAGC	176
PTGER3	chr1:71478094-71512579	CTTTTCTTCGCCTCTGCCTT	TCTTTCTGCTTCTCCGTGTG	290
PTGER4	chr5:40681909-81373848	ATCTTACTCATTGCCACCT	ATTTTTACTGACTTCTCGCT	114
PTGES	chr9:132502095-132515232	CACGCTGCTGGTCATC	AGGAAAAGGAAGGGGTAG	218
PTGES2	chr9:130883406-130884707	GTGGGTGGCTGCTGTGGG	TGCTCTGCGCGGGGACATT	195
PTGES3	chr12:57058139-57060035	GGTGGTGATGAGGATGT	GGGTTAAAGTAGGCAAATAC	214
PTGFR	chr1:78959186+78963629	CGATAATGTGTGTCTCCTGT	CTATGTAAGAAATCACTTTGCT	112
PTGIR[3]	chr19:47124350-47124424	GCCGATCAGCTGCTGTTTCT	TTTCCTCTGTCCCTCACTCTCTTC	75
PTGIS[2]	chr19:48140729-48156126	CCGTGGCTCCCTGTCAGT	GCAGCTTCCACAGGCGAC	72
TBXA2R	chr19:3595775-3599890	TGGGGATCATGGTGGTGG	CGGCGCGGCGGAACAGG	201

[1] Genome Browser Version Feb. 2009 (GRCh37/hg19)

[2] from reference [8] (references are listed in page 4)

[3] from reference [9]

Table S7. Primers used in this study for dissociation curve analysis and bisulfite sequencing of DNA methylation content.

Gene	Outer forward primer	Outer reverse primer	Inner forward primer	Inner reverse primer	Product size (bp)
AKR1B1	GAGTATTTATTTTTTAGGTATT	TTTCCCACCAAATACAACA	AATTTTAGGATGGGTATTTTG	AACTAAAAAACTCCTTTCTAC	325
HPGD	GGGTTTTTTTTTAGTTATTTTT	CTATCAACTACCTACCAAT	GGAGTGTGTGGGTAGAGAAA	AAACCAAAAACTAAAAAAAAACTT	403
PTGDR	ATTTTAGTAGTTTTTTTTTTGTTT	AACACCACCCCAACCCC	AAAACCAACAAATTACCCA	GAGTTTGTTTTTATTGAGAA	420
PTGDS	TTTTTTGGGAGGAGTGAG	ATTCTATACCCTAACTAAAC	GTTTTATATTTGGGTGTTAG	-	304
PTGER1	AAGAGGTTAGTAAGTAGTGT	AAACCTACCACTTCCTAA	GAAGAGGTTAGTAAGTAGTG	CCTACCACTTCCTAAAC	294
PTGER2	TAAGTGTTTTTGTATTTTTTTTTA	TCCAAAACCCCCCTTCCC	TTTTTGTATTTTTTTTTAGTTTTTT	ACTTACCCTAAATCTCCC	469
PTGER3	GAAAAGGTTGTTTTAGTTATG	TCACCATACTACTCACTAAT	TTGGTGTTGATGAAGTATTA	AATCCTTCCTACTATACATC	382
PTGER4	GGTTAATTTTAGGTAGAATT	CCTCTATACAAACTTTTCT	GGATAGGAGGTTTAAGAA	TTCTCCTCCTCCAAATT	365
PTGES	GTTTAGTAAGTAGTAGGTG	AACTACCATTATCAACTC	GTTTAGTAAGTAGTAGGTG	AACTACCATTATCAACTCC	395
PTGES2	TTATAAGTAGGTAGAGG	CTAAAAATTCCCTTAAC	GTTGATTAGTATTTTTATT	CCAAAATCCTAACCCTT	401
PTGES3	GTttagTaagTagtaggtg	cacaAACTACCATTATCAAC	gTaagTagtaggtgTtTaa	AACTACCATTATCAACTCC	383
PTGFR	TAGTATTGTTTTGTGTTTAT	TACATTAAAATCAACCTCT	GAGAGAAGAGGAAGAGG	AAATAATACCTTATCATCCC	297
PTGIR	GGTAAAGAGGATGAGTATGGA	AATAAATCACCTAAAATCAAAA	-	CCAACAAAAACATCTAAATAAAC	427
PTGIS_A[1]	GTTTTTTTTGTTAAGAAGGTGT	ATAAATAATTCCAAAACATAATCAAA	TTAAGAAGGTGTAAGGTGGG	AACACTCCCATCTATATAATAA	316
PTGIS_B[1]	TTTTAAARTGGGTTGGGGTGGG	CCTTCCCACCTTACACCTTCTTA	GGAATTTTATTTGGGAGTGGGTT	CACCTTCTTAACAAAAAAAAC	346
PTGS1	AGATTTAGGTGTATGGTATA	TTCATTTCCCAAACTCAAAA	TATGTTATAAGGGGTGYGAT	CTCTTTCTCCTTTTATATCC	321
PTGS2	GAGGTGAGAGTGTTTTAGAT	CATAAACTTAATTTTCAATCTTAT	GAGGTGAGAGTGTTTTAGAT	CATAAACTTAATTTTCAATCTTAT	476
TBXA2R	GTTGAGTAGGTAGATGTG	ATAACCTAAAAATCCCAAAA	TAGTTTGGTTGTGATTAGG	CCAAAAAACTATAAAATATCC	325

[1] from reference [8] (references are listed in page 4

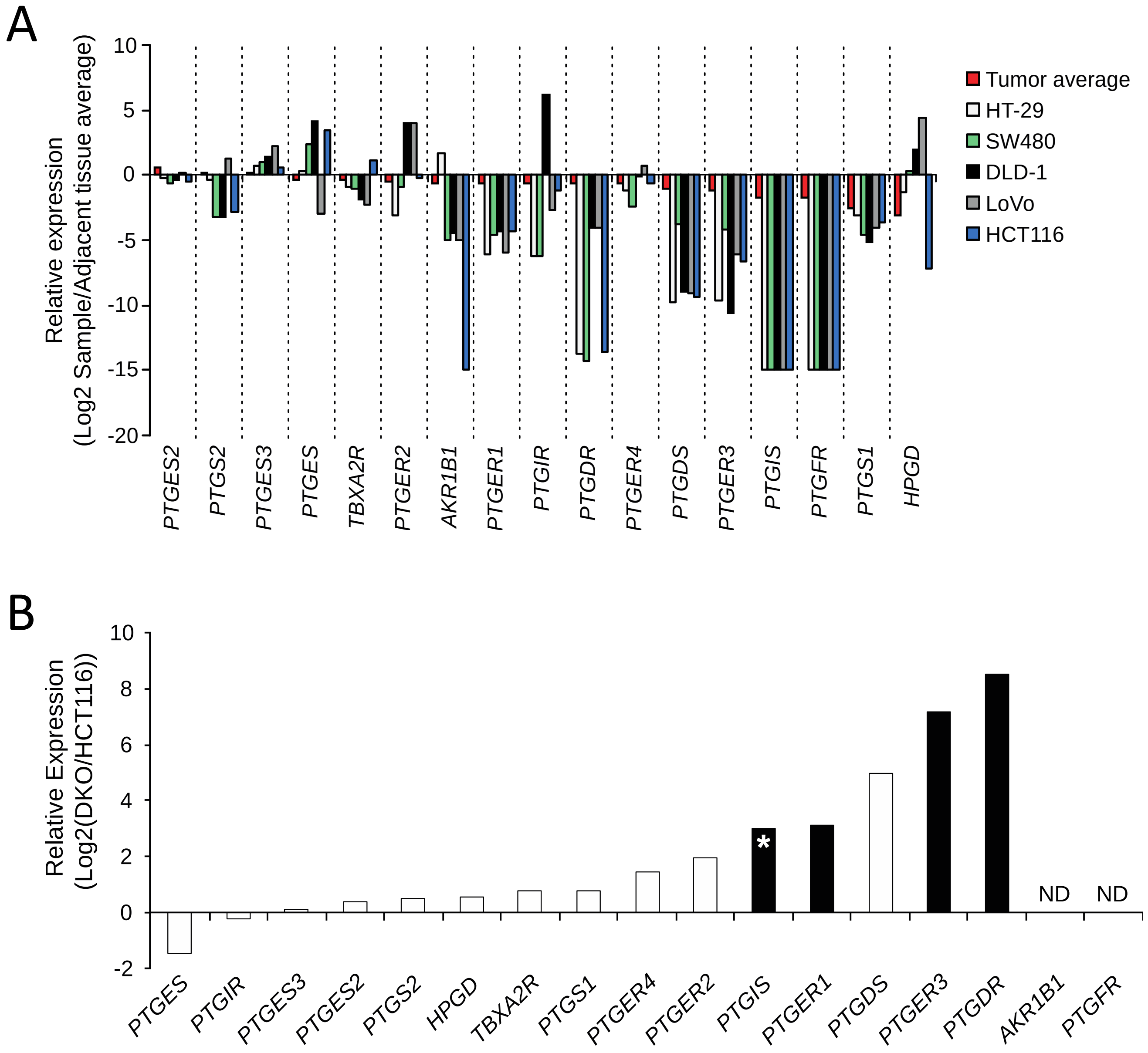

Figure S1. PTGS pathway expression profiling in CRC cell lines. (*A*) Expression profile of a panel of 5 colorectal cancer cell lines shows the same trends observed in primary colorectal tumor tissues (see **Figure 1**), being most transcripts downregulated or fully silenced. The only transcripts that keep or increase their expression levels are the PGE2 synthases (PTGES, PTGES2 and PTGES3). As a reference, we included the mean expression values for the tumor tissues analyzed by real time PCR (**Figure 1A**). (*B*) Gene expression was analyzed by qRT-PCR in HCT116 and its DNMT double knockout cell line, HCT116-DKO (referred to as DKO). Expression levels in DKO cells were normalized to HCT116. Black bars correspond to genes that undergo hypermethylation a large proportion of colorectal tumors and all of the 5 cell lines analyzed (see Figure 2*A-D*). *PTGIS* was only detected in DKO cells, not in HCT116, being the $\log_2$(DKO/HCT116) value arbitrary in this case (marked with a white *). No detectable levels of *AKR1B1* and *PTGFR*, which are fully methylated in HCT116, were found in HCT116, neither in DKO.

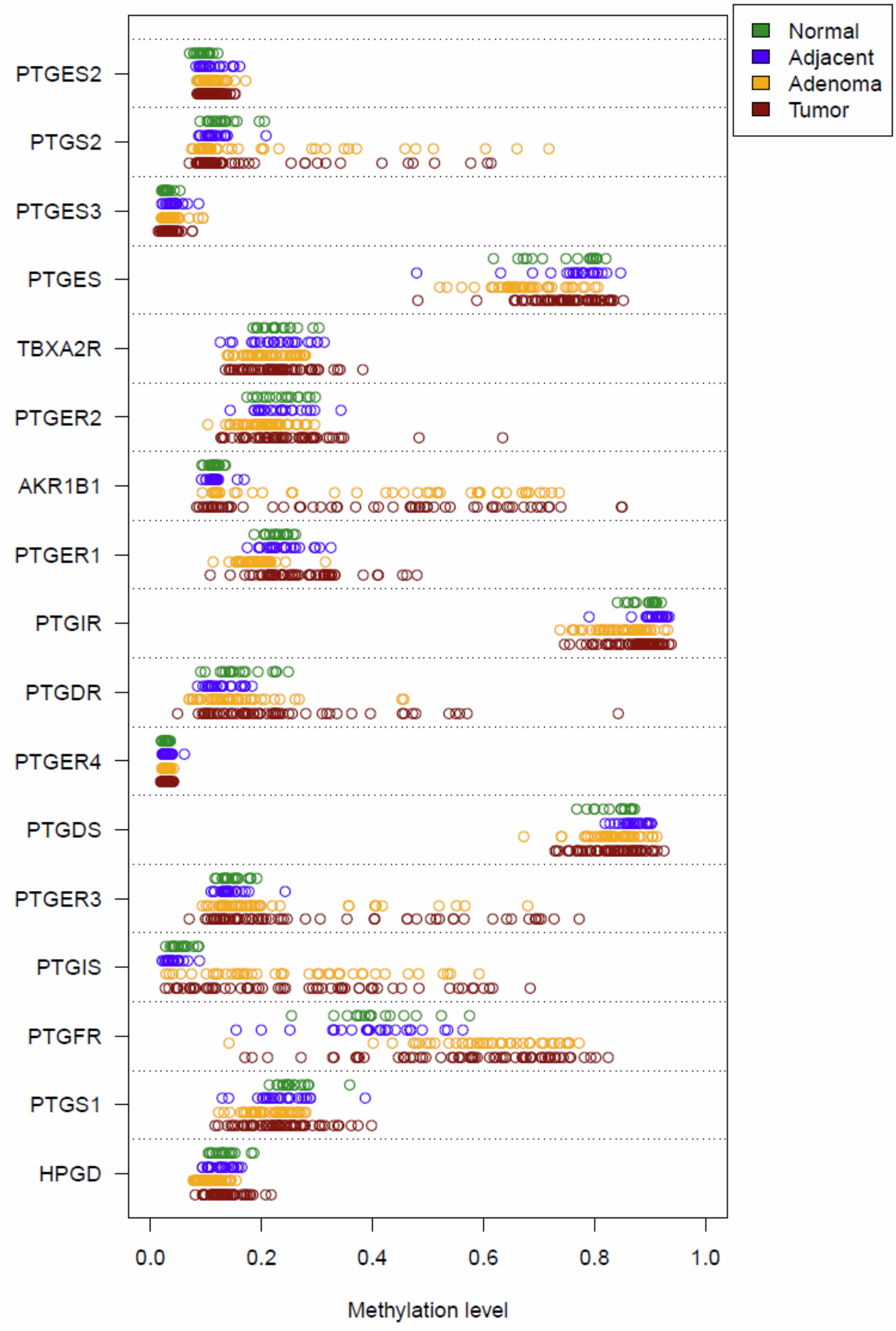

Figure S2. DNA methylation beta values quantified with InfiniumMethylation450K arrays for a cohort of 19 mucosae from healthy individuals (green), 22 adjacent normal tissues (blue), 42 adenomas (yellow) and 64 colorectal cancers. Normal mucosae from healthy and CRC patients display comparable profiles, while altered patterns affect in a similar way adenomas and carcinomas. Data were obtained from Luo et al. (supplementary reference 7).

A

PTGS pathway genes

	PTGIS	*AKR1B1*	*PTGFR*	*PTGER3*	*PTGER1*	*PTGDR*	*PTGS2*	*TBXA2R*	*PTGER2*	*PTGER4*	*PTGS1*	*PTGES*	*PTGES2*	*PTGES3*	*HPGD*	*PTGIR*
Mutation	13%	2%	2%	2%	1%	<1%	2%	<1%	<1%	1%	2%	0%	0%	<1%	1%	2%
Deleterious mutation	0%	2%	2%	2%	1%	0%	2%	0%	0%	1%	2%	0%	0%	0%	0%	0%

B

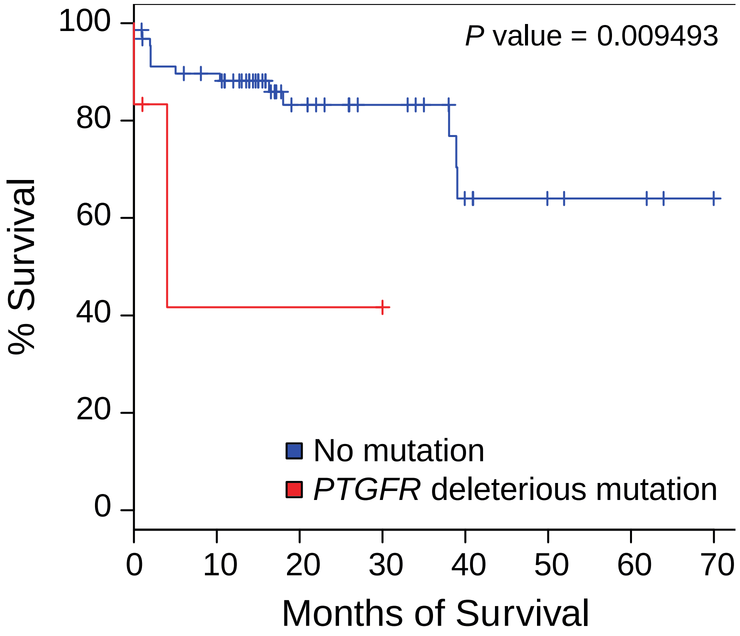

Figure S3: Mutations in PTGS pathway genes in CRC. (*A*) Mutation al rates of PTGS pathway genes in CRC (data obtained from the TCGA database). Deleterious mutation definition based on TCGA criteria. (*B*) Survival plot for CRC patients with and without deleterious mutations in *PTGFR*. Survival is greatly affected by the presence of deleterious mutations in this gene, which is frequently hypermethylated in colorectal tumors.

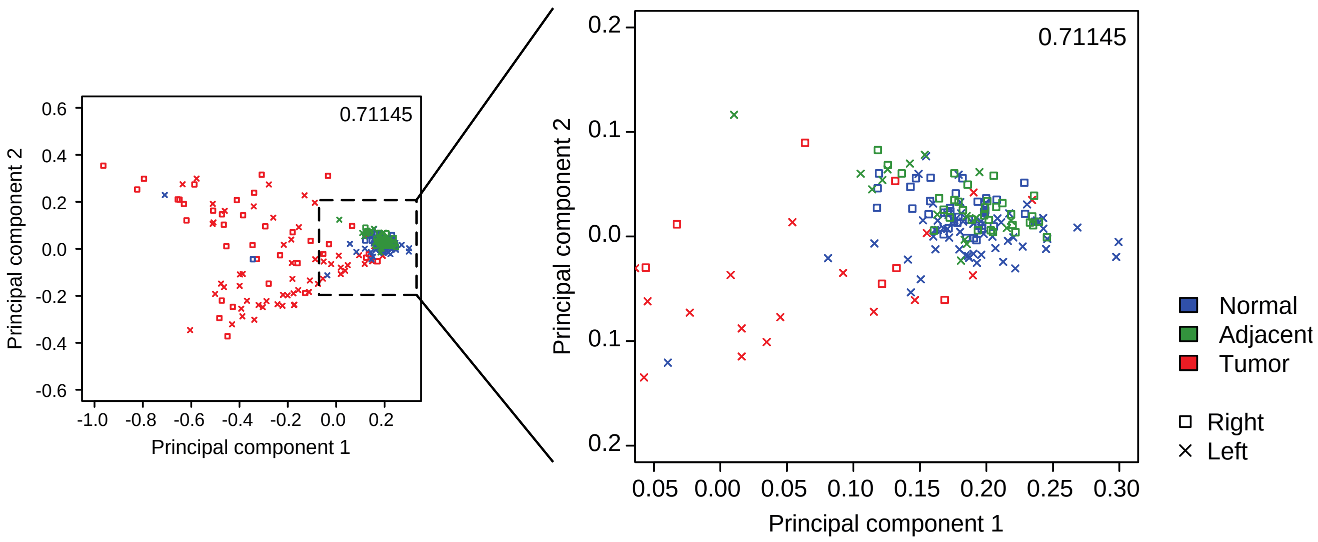

Figure S4: Principal component analysis of DNA methylation profiles in genes of the COX pathway in normal mucosa from healthy individuals, and the adjacent normal mucosa and tumor sample of patients with colorectal cancer. Samples from the left and right colon are depicted with distinct markers.

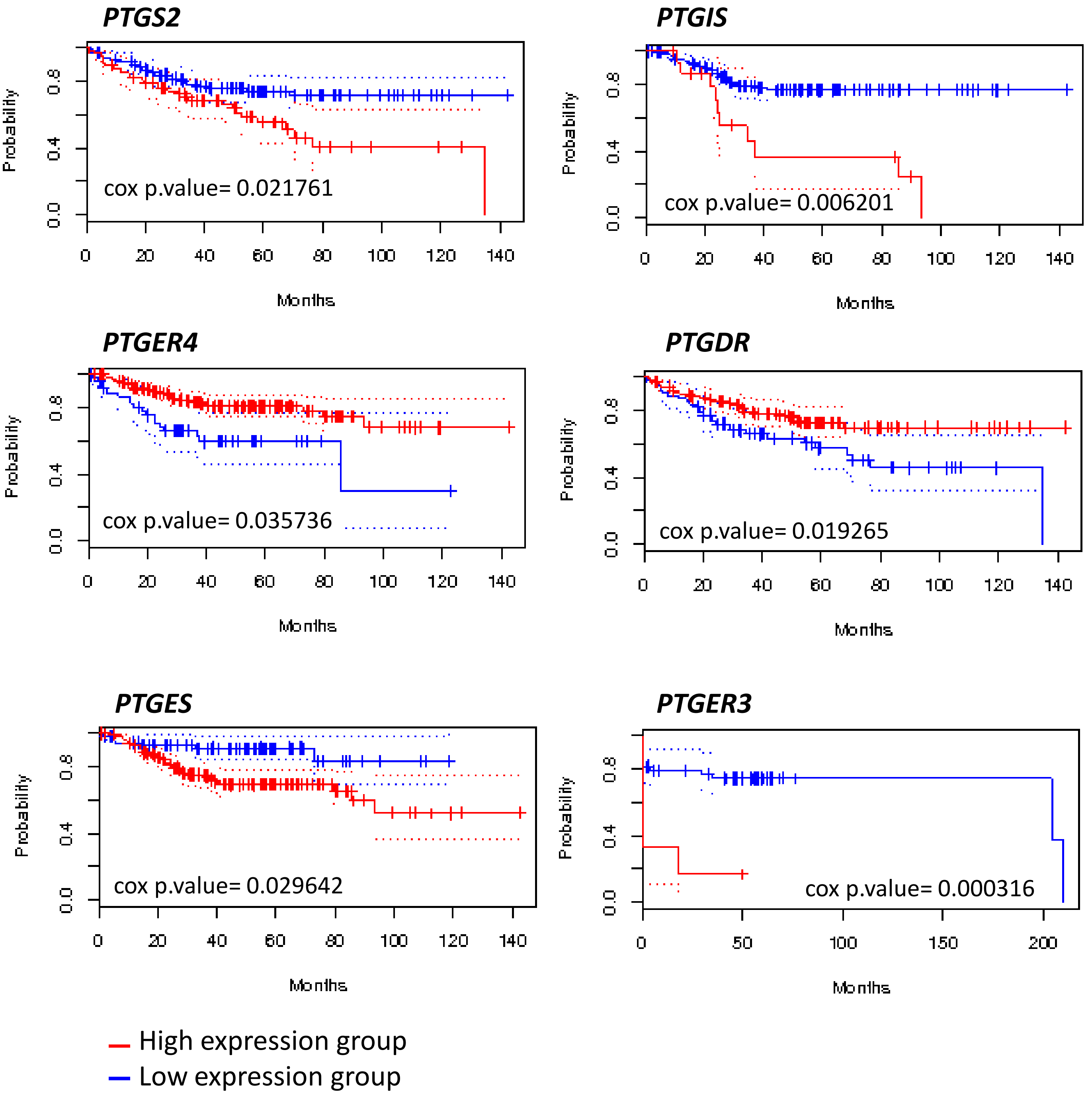

Figure S5. Survival analysis of CRC patients according to gene expression levels. Data were obtained from four datasets (GEO: GSE14333, GSE12945, GSE17536, GSE17537) with follow-up information available to analyze the survival rate depending on gene expression. The PrognoScan algorithm (Mizuno et al, 2009, supplementary reference 10) was used to classify the patients into either the High or Low expression group (minimum p-value approach). Survival curves were constructed using the Kaplan-Meier method.
